# Twist-mediated PAR1 induction is required for breast cancer progression and metastasis by inhibiting Hippo pathway

**DOI:** 10.1038/s41419-020-2725-4

**Published:** 2020-07-09

**Authors:** Yifan Wang, Ruocen Liao, Xingyu Chen, Xuhua Ying, Guanping Chen, Mingqian Li, Chenfang Dong

**Affiliations:** 1 Department of Pathology and Pathophysiology, and Department of Surgical Oncology (breast center) of the Second Affiliated Hospital, Zhejiang University School of Medicine, Hangzhou, China; 2 Cancer Institute of Integrative Medicine, Zhejiang Academy of Traditional Chinese Medicine, Tongde Hospital of Zhejiang Province, Hangzhou, China; 3 Zhejiang Key Laboratory for Disease Proteomics, Zhejiang University School of Medicine, Hangzhou, China

**Keywords:** Breast cancer, Epithelial-mesenchymal transition

## Abstract

Breast cancer is considered to be the most prevalent cancer in women worldwide, and metastasis is the primary cause of death. Protease-activated receptor 1 (PAR1) is a GPCR family member involved in the invasive and metastatic processes of cancer cells. However, the functions and underlying mechanisms of PAR1 in breast cancer remain unclear. In this study, we found that PAR1 is highly expressed in high invasive breast cancer cells, and predicts poor prognosis in ER-negative and high-grade breast cancer patients. Mechanistically, Twist transcriptionally induces PAR1 expression, leading to inhibition of Hippo pathway and activation of YAP/TAZ; Inhibition of PAR1 suppresses YAP/TAZ-induced epithelial-mesenchymal transition (EMT), invasion, migration, cancer stem cell (CSC)-like properties, tumor growth and metastasis of breast cancer cells in vitro and in vivo. These findings suggest that PAR1 acts as a direct transcriptionally target of Twist, can promote EMT, tumorigenicity and metastasis by controlling the Hippo pathway; this may lead to a potential therapeutic target for treating invasive breast cancer.

## Introduction

Breast cancer is a heterogeneous disease. Despite extensive progress in understanding mechanisms of breast cancer progression and developing therapeutic strategies, ~90% of breast cancer mortality is due to recurrent and metastasis^1^. The increased motility and invasive capabilities of metastatic tumor cells are associated with the epithelial-mesenchymal transition (EMT), a phenotypic conversion by which cells lose apico-basal polarity and acquire highly motile mesenchymal properties^2–6^. EMT confers tumor cells with cancer stem cell (CSC) features, promoting tumor progression and metastasis^7^. Interestingly, increasing studies have shown that some highly invasive breast cancer cells possess many EMT markers and CSC-like properties^2,8–11^.

The Hippo pathway is a key player in several carcinogenesis processes, including cell proliferation, apoptosis, and stem cell maintenance^12,13^. The core upstream components of the Hippo pathway such as MST1/2, SAV1, and Lats1/2 are tumor suppressors, whereas the downstream components YAP/TAZ and TEADs are oncogenes^14^. MST1/2 kinases together with the SAV1, phosphorylate and activate the LATS1/2; activated LATS1/2 kinases phosphorylate and inactivate the transcription co-activators YAP/TAZ^15^. Loss of Hippo kinases or induction of YAP/TAZ activates TEAD-mediated target gene transcription, resulting in induction of EMT and maintenance of CSC properties^16–18^. Recent studies show that the Protease-activated receptor-1 (PAR1) is an upstream signal of Hippo pathway^19,20^. PAR1, also known as coagulation factor II receptor (F2R), is a seven-transmembrane G-protein-coupled receptor (GPCR) family member involved in the invasive and metastatic processes of cancer cells^21–23^. PAR1 inhibits kinases Lats1/2 through the G12/13 and Rho GTPase, leading to activation of YAP/TAZ by decreasing its phosphorylation and increasing nuclear localization^19^. A better understanding of the functions and mechanisms of PAR1 will contribute to the development of new therapeutic strategies.

In this study, we report that PAR1 expression is elevated in breast cancer with high invasive ability and predicts poor prognosis. PAR1 expression is transcriptionally upregulated by Twist. Knockdown of PAR1 expression inhibits EMT and reduces the tumorigenic and metastatic potential of breast cancer cells through activating Hippo pathway. Our study reveal a critical mechanism and function of how PAR1 contributes to EMT and metastasis in aggressive breast cancer.

## Materials and methods

### Plasmids, siRNA, and antibodies

PAR1 shRNA was purchased from Sigma-Aldrich (MISSION shRNA Plasmid DNA). Human Twist was amplified from a HeLa cDNA library, and then subcloned into the lentiviral vector pLenti6.3⁄V5. Human PAR1 was amplified from the SUM159 cDNA and subcloned into pLVX. Antibodies for E-cadherin (610181), N-cadherin (610920) and TAZ (560235) were purchased from BD Transduction Laboratories. Antibody for Vimentin (ms-129-p) was from Thermo Scientific. Antibodies for Twist (sc-81417), PAR1 (sc-13503), YAP (sc-15407) and CTGF (sc-14939) were from Santa Cruz Biotechnology.

### Chemicals and inhibitors

Human Alpha Thrombin (HT 1002a) was obtained from Enzyme Research Laboratories. Thrombin receptor activator peptide 6 (TRAP6, S1820) was purchased from Sigma-Aldrich. Vorapaxar (HY‑10119) was obtained from MedChemExpress. Botulinum toxin C3 (CT04) was purchased from Cytoskeleton, Inc.

### Cell culture

T47D cells were grown in RPMI1640 plus 10% FBS. MCF7, MDA-MB-231 and Bs578T cells were grown in DMEM supplemented with 10% FBS. SUM159 cells were grown in Ham’s F-12 nutrient mixture supplemented with 5% FBS, 5 μg/mL insulin, 10 ng/mL EGF. For establishing stable clones, transfected cells were selected with 1 μg/mL puromycin for 4 week.

### Quantitative real-time PCR

Total RNA was extracted using the RNeasy Mini kit (Qiagen) according to the manufacturer’s instructions. Quantitative real-time PCR assays were performed using LightCycler 480 SYBR Green I Master following manufacturer’s protocol (Roche).

### Luciferase reporter assay

Luciferase reporter assays were performed following the protocol previously described^24,25^. Cells were plated in 24-well plates and transfected with promoter-luciferase plasmid along with pcDNA3 or pcDNA-Twist in each well using X-tremeGENE 9 (Roche). To normalize transfection efficiency, cells were co-transfected with the Renilla luciferase vector (pTK-RL). Forty-eight hours after transfection, luciferase activity was assessed using the Dual-Luciferase Reporter Assay System (Promega). Three independent experiments were performed, and the calculated means and standard deviations are presented.

### ChIP

ChIP assays were carried out as described previously^24,26^. The primers used for PAR1 promoter were: 5′-TGAGTCACTGACAGCTTCGC-3′ and 5′-AGTGAGAGTCTCTGCGCTGG-3′; 5′-CTGAGCCAGGGAGATCGAG-3′ and 5′-AGGAGAGAAATGGCCTGGTA-3′; 5′-AAGTGTCCGGGCTCTAGTGG-3′ and 5′-TGCGGGCATATTCGGAGTTC-3′. ChIP assays were performed using the Imprint ChIP kit according to the manufacturer’s instructions (Sigma-Aldrich).

### Flow cytometry

Cells were detached using trypsin-EDTA (Hyclone), resuspended in growth medium and counted. Cells were washed twice and resuspended in 100 μL PBS and then stained with 2 μL of each antibody: CD44-PE/Cy7 and CD24-PE (eBiosciences). Cells were labeled on ice for 30 min in the dark prior to washing and analysis. After two wash steps with PBS, the stained cells were suspended in 500 μL PBS, and detected by BD FACS Canto II.

### Colony formation assay

Colony formation assay was performed using a double-layer soft agar system (a bottom layer of 0.7% agar and a top layer of 0.35% agar) in 24-well plates. Cells were suspended in culture medium with 0.35% agar and laid on the top of the base agar layer (0.7% agar) in 24-well plates and incubated at 37 °C and 5% CO_2_ for 15–20 days. At the end of the incubation time, the colonies were stained with P-iodonitrotetrazolium violet and counted.

### Migration assay and invasion assay

Migration and invasion assays were performed as previously described^24,26^. For invasion assay, cancer cells were placed on top of the Matrigel in the upper chamber, while the bottom chamber was filled with culture medium plus 10 ng/mL EGF as the chemo-attractant. The migrated and invaded cells, on the underside of the Boyden chamber membrane, were stained with crystal violet and counted with a microscope. All experiments were performed in triplicate.

### Tumorsphere assay

Tumorsphere assays were performed as previously described^27,28^. In brief, cell monolayers were plated as single-cell suspensions on ultra-low attachment 6-well plates (Corning) in DMEM/F12 medium containing 5 mg/mL insulin, 20 ng/mL EGF, 0.5 mg/mL hydrocortisone and 2% B27. After 7–14 day incubation, the presence of spheres was assessed using an inverted microscope.

### Tumorigenesis assay and lung metastasis model

All procedures on animals were performed according to protocols approved by the Institutional Animal Care and Use Committee at Zhejiang University. To evaluate the effect of PAR1 on tumorigenesis, female SCID mice (6–8-week old) were injected with breast cancer MDA-MB-231/SUM159 cells via mammary fat pad, and mice were randomly divided into two groups: vector control and stable clones with PAR1-knockdown expression (1 × 10^6^ cells per mouse for MDA-MB-231 and 5 × 10^6^ cells per mouse for SUM159; six mice/group). Tumor formation was monitored every 2 to 4 days for a 4-week period. Tumors’ size and weight were measured. To examine the effect of PAR1 on tumor metastasis, SCID mice were randomly divided into two groups and injected with MDA-MB-231 cells with stable control vector or knockdown of PAR1 expression via tail vein (1 × 10^6^ cells per mouse; six mice/group). No blinding was applied in these experiments. Four weeks later, lung metastasis was analyzed by IVIS Lumina XRMS (PE). After mice were sacrificed, the lung tissues were fixed with 10% formalin, paraffin embedded, stained with hematoxylin and eosin, and examined under microscopy. Data were analyzed by Student’s *t* test; *P* < 0.05 was considered significant.

### Statistical analysis

Experiments were repeated at least three times. Results are presented as mean ± SD or SEM. A two-tailed Student’s *t* test was used to compare two groups. Correlations between PAR1 and Twist were analyzed by Pearson’s correlation method and Spearman’s rank correlation test. Survival curves were generated using the Kaplan–Meier method, and differences were evaluated by the log-rank test. For all statistical tests, *P* < 0.05 was considered statistically significant. All statistical tests are justified as appropriate and the data meet the assumptions of the tests. The variance is similar between the groups that are being statistically compared.

## Results

### PAR1 expression is upregulated in highly invasive breast cancer

**Fig. 1:**
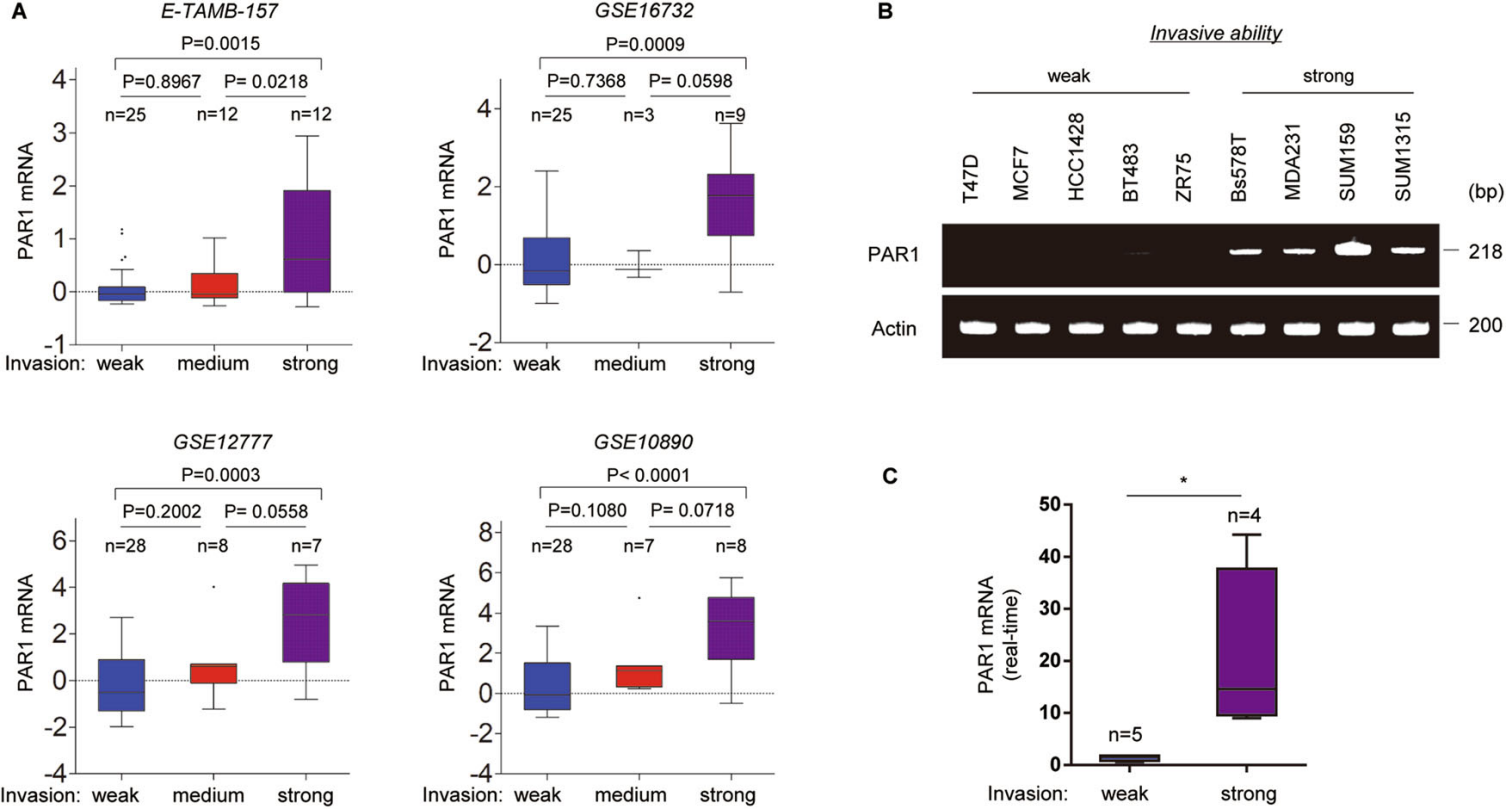
Elevated expression of PAR1 highly correlated with invasive breast cancer cells. **a** Box plots and bar charts indicated PAR1 mRNA expression in different invasion abilities of breast cancer cells from four gene expression datasets (E-TAMB-157, GSE16732, GSE12777 and GSE10890). **b**, **c** Expression of PAR1 mRNA was analyzed by either semi-quantitative RT-PCR (**b**) or quantitative real-time PCR (**c**) in a representative panel of breast cancer cell lines (MDA-MB-231 is abbreviated to MDA231). Data are shown as mean ± SD based on three independent experiments. *P < 0.01 by Student’s *t* test.

To explore the role of PAR1 in breast cancer, we systematically analyzed PAR1 expression in four gene expression datasets (E-TAMB-157, GSE16732, GSE12777, and GSE10890), which contain 49, 37, 43, and 43 breast cancer cell lines, respectively^29–31^. We noticed that PAR1 expression was significantly higher in highly invasive breast cancer cells than in low invasive breast cancer cell lines (Fig. 1a and Supplementary Fig. 1a). To confirm this observation, we detected the PAR1 mRNA level by semi-quantitative PCR and quantitative real-time PCR in a representative panel of human breast cancer cell lines, including five low invasive and four highly invasive cell lines^32^. Consistently, PAR1 mRNA expression was remarkably upregulated in highly invasive breast cancer cell lines (Fig. 1b, c and Supplementary Fig. 1b). Our data indicate that PAR1 overexpression is restricted to highly invasive breast cancer cells.

### PAR1 positively correlates with Twist and is a direct target of Twist

**Fig. 2:**
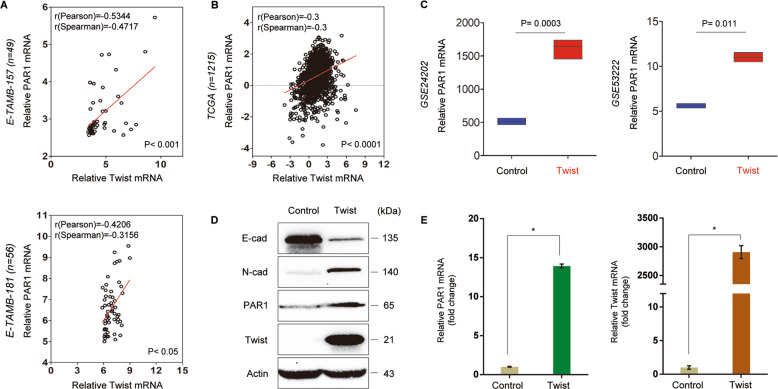
PAR1 positively correlates with Twist expression. **a**, **b** Analysis of E-TAMB-157, E-TAMB-181 (**a**) and TCGA (**b**) datasets for the expression of PAR1 and Twist. The relative level of PAR1 was plotted against that of Twist. Correlations were analyzed using Pearson’s correlation method and Spearman’s rank correlation test. **c** Analysis of GSE24202 and GSE53222 datasets for PAR1 mRNA expression in HMLE and T47D cells with or without Twist expression. **d** Expression of PAR1, Twist, E-cadherin, and N-cadherin was examined by Western blotting in T47D cells transfected with control vector or Twist-expressing vector, and actin was served as a loading control. Representative images were presented from three independent experiments. **e** Expression of PAR1 and Twist was analyzed by quantitative real-time PCR in T47D cells transfected with control vector or Twist-expression vector. Data are presented as mean ± SD of three separate experiments, *P < 0.01 by Student’s *t* test.

To determine potential functions and mechanisms of PAR1 in breast cancer, we then explored the correlation of PAR1 with other molecules. Co-expression analysis of PAR1 with other genes in two gene expression datasets (E-TAMB-157 and E-TAMB-181) showed that PAR1 expression positively correlated with Twist expression (Fig. 2a). A similar result was found in analyzing another large gene expression dataset (TCGA) that contains 1215 breast cancer patients (Fig. 2b). To investigate the causal association of PAR1 with Twist, we analyzed PAR1 expression in HMLE and T47D cells with ectopic Twist expression in two previous datasets (GSE24202 and GSE53222)^33,34^, displaying striking upregulation of PAR1 expression by Twist (Fig. 2c). Next, we established stable clones with empty control vector or Twist expression in T47D cells. As expected, Twist expression significantly downregulated E-cadherin expression and upregulated N-cadherin expression in T47D cells (Fig. 2d), which is strongly indicative of EMT. Interestingly, Twist expression remarkably induced PAR1 expression in both mRNA and protein levels (Fig. 2d, e). These results suggest that Twist as a transcriptional activator may induce PAR1 expression via transcriptional regulation.

**Fig. 3:**
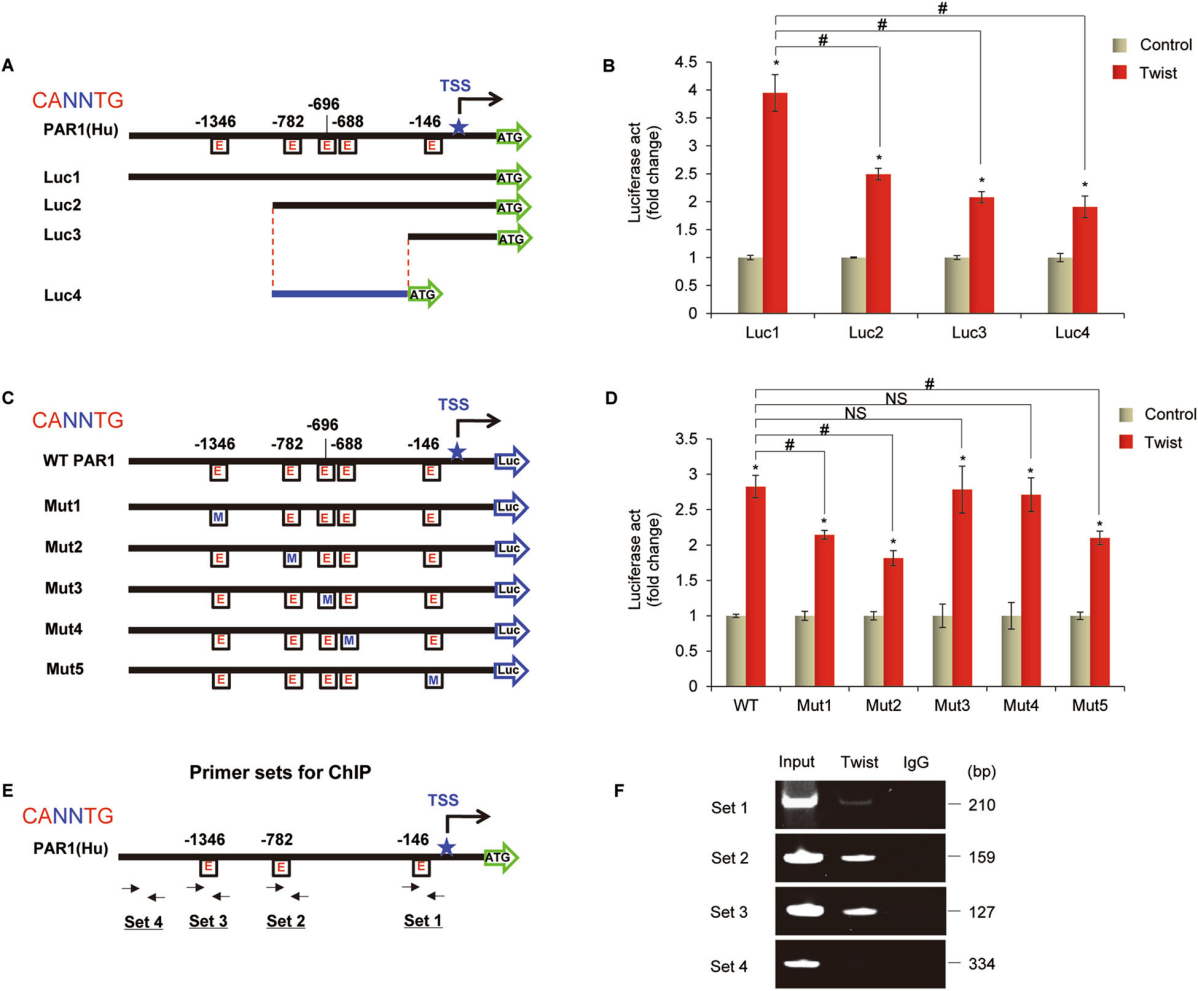
PAR1 is a direct transcriptional target of Twist. **a** A scheme showing positions of five potential Twist-binding E-boxes on the PAR1 promoter and PAR1 promoter-luciferase construct used. E, E-box. **b** PAR1 promoter luciferase constructs (Luc1, Luc2, Luc3, and Luc4) were co-expressed with Twist or vector in HEK293T cells. After 48 h, luciferase activities were determined and normalized (mean ± SD of three separate experiments), *and #, *P* < 0.01 by Student’s *t* test. **c** A scheme showing positions of potential Twist-binding E-boxes on the PAR1 promoter. PAR1 promoter luciferase construct and mutated derivatives were also shown. E, E-box; M, mutated. **d** PAR1 promoter luciferase construct (WT) as well as its mutants (Mut1, Mut2, Mut3, Mut4, and Mut5) were co-expressed with control vector or Twist-expressing vector in HEK293T cells. Luciferase activities were analyzed as in (**b**). Data are shown as mean ± SD based on three independent experiments, * and #, *P* < 0.01 by Student’s *t* test; NS, no significance. **e** Positions of four sets of primers in the PAR1 promoter for the ChIP assays were shown. **f** ChIP analysis for binding of Twist to the PAR1 promoter in T47D-Twist cells.

Given the immediate induction of PAR1 expression by Twist, we then determined whether PAR1 expression was regulated directly by Twist. We noticed that PAR1 promoter contained five potential Twist-binding E-boxes (CANNTG) from −1346 bp to transcription start site (TSS) (Fig. 3a). To investigate whether these E-boxes are critical for Twist-mediated gene transcription, we cloned the human PAR1 promoter (Luc1 = −1496 to +265 bp) and generated several deletion mutants of promoter-luciferase constructs according to the location of these E-boxes (Fig. 3a). By expressing a full-length PAR1 promoter (Luc1) in HEK293T cells, Twist significantly activated PAR1 promoter activity (Fig. 3b). The Luc2 without the region between −1496 bp and −998 bp partially lost the reporter activity (Luc2 vs Luc1), indicating that E-boxes at −1346 bp was important for Twist-mediated PAR1 activation (Fig. 3b). Three E-boxes between −998 bp and −204 bp as well as E-box at −146 bp were also important for Twist-mediated PAR1 activation, as deletion constructs (Luc3 and Luc4) without either region still remained low reporter activation to respond to Twist expression (Fig. 3b). To pinpoint the exact binding E-boxes, several constructs with point mutants were created in the potential Twist-responsive E-boxes (Mut1-Mut5) (Fig. 3c). Mut1, Mut2 and Mut5 significantly reduced the reporter activity induced by Twist, suggesting that E-boxes at −1346, −782, and −146 bp are required for Twist-induced transcriptional activation (Fig. 3d). To further examine whether Twist directly binds to the PAR1 promoter, we performed chromatin immunoprecipitation (ChIP) by using four sets of primers (Fig. 3e). We found that Twist bound to the PAR1 promoter in T47D cells with Twist expression (Fig. 3f). These data confirm that PAR1 is a direct target of Twist.

### PAR1 alters the expression of EMT markers and inhibits the Hippo pathway

**Fig. 4:**
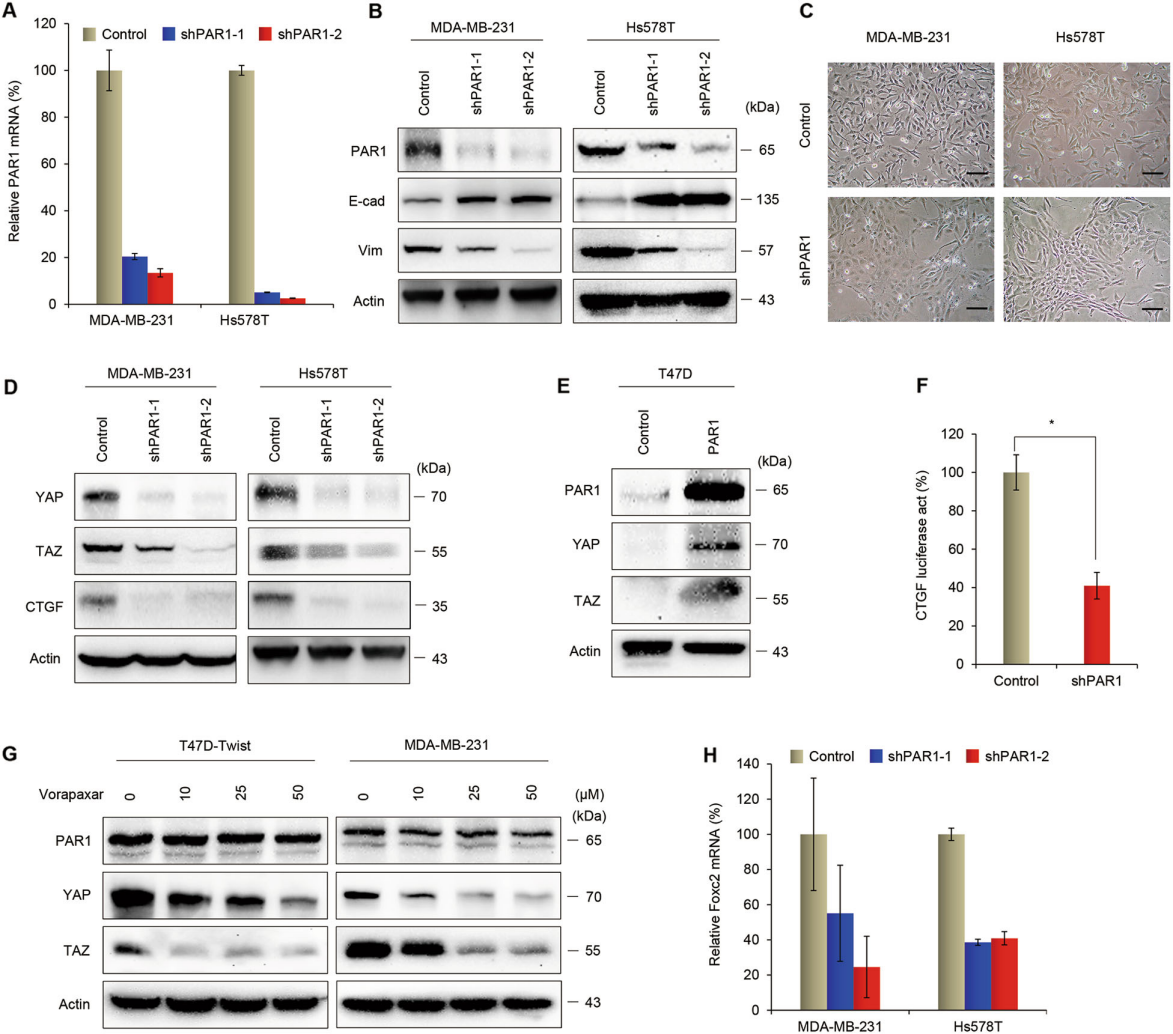
PAR1 alters the expression of EMT markers and inhibits the Hippo pathway. **a** Expression of PAR1 was analyzed by quantitative real-time PCR in MDA-MB-231 and Hs578T cells with stable control vector or knockdown of PAR1 expression. Data are presented as mean ± SD of three separate experiments. **b** Expression of PAR1, E-cadherin, and Vimentin was assessed by western blotting in MDA-MB-231 and Hs578T cells with stable control vector or knockdown of PAR1 expression, and actin was served as a loading control. Representative images were presented from three independent experiments. **c** The morphologic changes of MDA-MB-231 and Hs578T cells with stable control vector or knockdown of PAR1 expression were shown in representative images. Scale bars, 100 μm. **d** Expression of YAP, TAZ, and CTGF was analyzed by western blotting in MDA-MB-231 and Hs578T cells with stable control vector or knockdown of PAR1 expression. Representative images were presented from three independent experiments. **e** Expression of PAR1, YAP, and TAZ was assessed by western blot in T47D cells transfected with control vector or PAR1-expressing vector, and actin was served as a loading control. Representative images were presented from three independent experiments. **f** The CTGF reporter activity was examined in MDA-MB-231 cells with control vector or knockdown of PAR1 expression. After 48 h, luciferase activities were determined and normalized (mean ± SD of three separate experiments) *P < 0.01 by Student’s *t* test. **g** T47D-Twist and MDA-MB-231 cells were serum-starved for 24 h, and then treated with indicated concentration of vorapaxar. Expression of PAR1, YAP, and TAZ was assessed by western blotting, and actin was served as a loading control. Representative images were presented from three independent experiments. **h** Expression of Foxc2 was analyzed by quantitative real-time PCR in MDA-MB-231 and Hs578T cells with stable control vector or knockdown of PAR1 expression. Data are presented as mean ± SD of three separate experiments.

In order to study the function and molecular mechanism of PAR1, we created stable transfectants with control vector or knockdown of PAR1 expression in two mesenchymal-like cell lines MDA-MB-231 and Hs578T (Fig. 4a, b). Because PAR1 is a direct transcriptional target of Twist that also plays key roles in induction of EMT, we first assessed whether a correlation exists between PAR1 expression and the EMT process in these cells. Loss of epithelial molecules and the acquisition of mesenchymal markers are regarded as fundamental events in EMT^2,4^. Downregulation of PAR1 caused a dramatic increase in the expression of epithelial molecule (E-cadherin) and a significant decrease in the expression of mesenchymal marker (Vimentin) (Fig. 4b). Importantly, knockdown of PAR1 expression in MDA-MB-231 and Hs578T cells converted the mesenchymal morphology to an epithelial phenotype 10–12 days after shPAR1 lentiviral infection (Fig. 4c). These data indicate a critical role for PAR1 in the induction of EMT in breast cancer cells.

Recent research has identified various upstream components of the Hippo pathway, including cell polarity, mechanotransduction and GPCR signaling^35^. Since PAR1 inhibits the Lats1/2 kinases, we reasoned that Twist-induced PAR1 expression might increase YAP and TAZ activity. Indeed, YAP and TAZ expression was upregulated in T47D-Twist cells (Supplementary Fig. 2a). We noticed that mRNA expression levels of several well-known YAP/TAZ target genes, including CTGF, ANKRD1, Cyr61, and BIRC5, were also increased (Supplementary Fig. 2b). Knockdown of Twist expression decreased PAR1 expression and activity in Hs578T cells (Supplementary Fig. 2c). In addition, PAR1 knockdown in MDA-MB-231 and Hs578T cells caused a decrease, whereas exogenous PAR1 expression in T47D cells resulted in an increase in both YAP and TAZ expression (Fig. 4d, e and Supplementary Fig. 2d). To further evaluate the functional role of PAR1 in modulating the Hippo pathway, we examined the CTGF promoter luciferase activity. Significantly, knockdown of PAR1 expression decreased the activity of CTGF reporter (Fig. 4f). We next investigated whether pharmacological inhibition of PAR1 could regulate the Hippo pathway in breast cancer cells. Vorapaxar is a highly specific, virtually irreversible PAR1 antagonist that has been approved for clinical use^36^. We found that vorapaxar efficiently blocked the expression of YAP and TAZ in T47D-Twist and MDA-MB-231 cells (Fig. 4g). PAR1 is also known as the thrombin receptor^37^. Our data showed that the addition of thrombin did not cause a change in the concentration of PAR1 in breast cancer cells (Supplementary Fig. 3a). We then examined the effect of a selective PAR1-activating peptide (TRAP6) and a specific inhibitor of Rho GTPase (botulinum toxin C3) on YAP and TAZ expression. We found that addition of TRAP6 resulted in induction of YAP and TAZ expression in T47D-Twist cells, whereas the treatment of botulinum toxin C3 efficiently block the effect of TRAP6 (Supplementary Fig. 3b). These results suggest that Twist-induced activation of PAR1 might trigger the activation of Rho GTPase, leading to the induction of YAP/TAZ. To gain insight into the molecular mechanism underlying PAR1-induced EMT, we further measured the mRNA level of transcription factor Foxc2 that has been shown to activate EMT program. Strikingly, Foxc2 expression was decreased in MDA-MB-231 and Hs578T cells with knockdown of PAR1 expression (Fig. 4h). Taken together, these results indicate that downregulation of PAR1 activates the Hippo pathway and thus inhibits the expression of YAP/TAZ, leading to a decrease of Foxc2 and suppression of EMT in breast cancer cells.

### PAR1 enhances CSC properties and is required for tumorigenicity in breast cancer cells

**Fig. 5:**
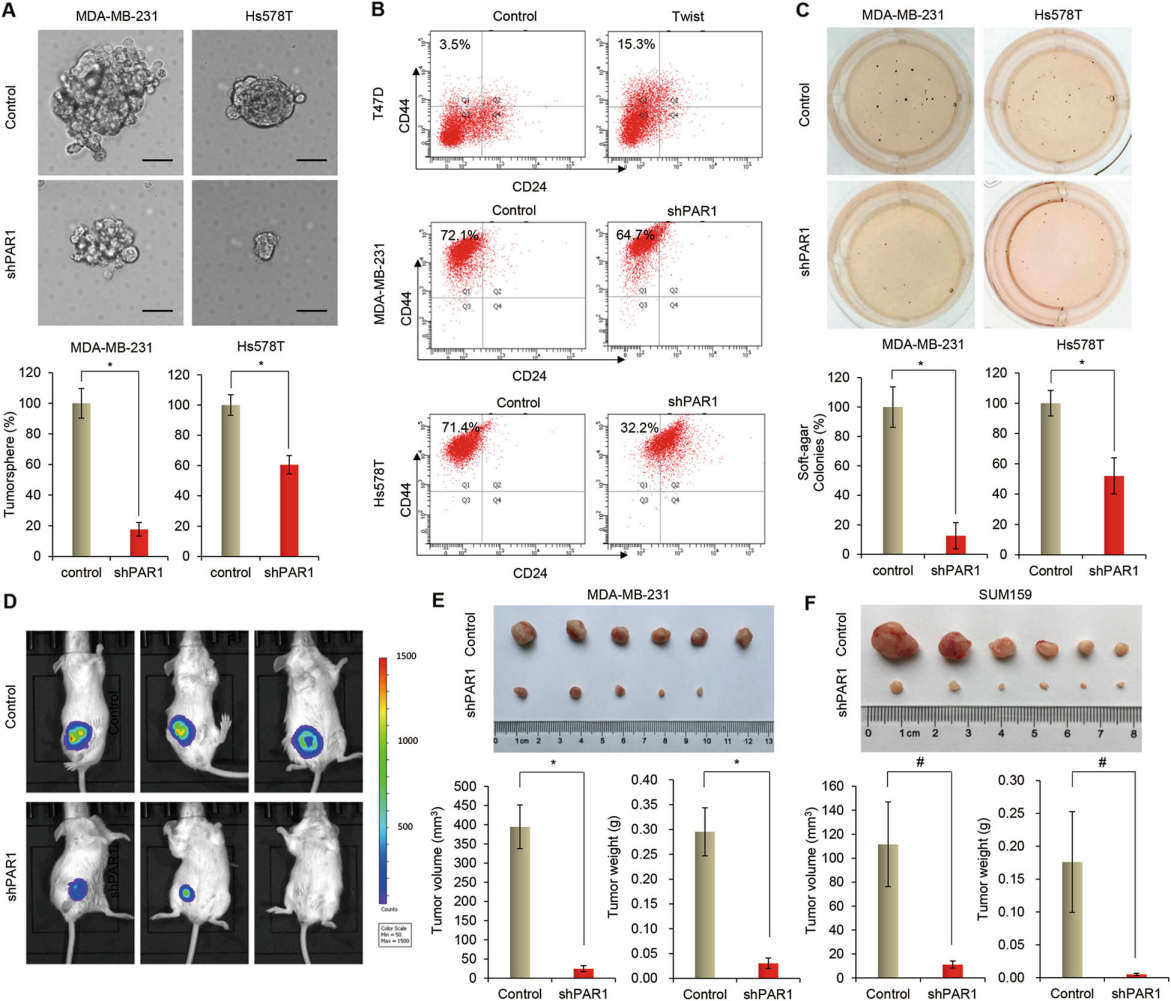
Knockdown of PAR1 expression inhibits tumorigenicity in vitro and in vivo. **a** Tumorsphere formation was analyzed in MDA-MB-231 and Hs578T cells with stable control vector or knockdown of PAR1 expression (bottom panel). Representative images of tumorspheres were shown (upper panel). Scale bars, 50 μm. Data are presented as a percentage of control vector cell lines. *P < 0.01 by Student’s *t* test. **b** Representative images showed populations of CSCs (CD44^high^/CD24^low^) analyzed by flow cytometry in T47D cells with stable control vector or Twist-expression vector as well as MDA-MB-231 and Hs578T cells with stable control vector or knockdown of PAR1 expression. **c** Soft-agar assay was performed using MDA-MB-231 and Hs578T cells with stable control vector or knockdown of PAR1 expression (bottom panel). Representative images of colony formation are shown (upper panel). Data are presented as a percentage of control vector cell lines (mean ± SD of three separate experiments) *P < 0.01 by Student’s *t* test. **d**–**f** MDA-MB-231 cells (**d**–**e**) SUM159 cells (**f**) with stable control vector or knockdown of PAR1 expression were injected into the mammary fat pad of SCID mice. The size of tumor was recorded after 4 week. Tumor volume and weight were also measured. Data are represented as a mean ± SEM from six mice. #*P* < 0.05 and *P < 0.01.

Having identified the association of PAR1-mediated EMT process with Hippo signaling in invasive breast cancer cells, we then assessed the functional role of PAR1 in vitro and in vivo. Accumulating evidence has shown that EMT confers tumor cells with CSC properties, contributing to tumor initiation and progression^2,38^. As PAR1 is overexpressed in highly invasive breast cancer cells and involved in the EMT process, we speculated that PAR1 expression might endow stem-like properties to invasive breast cancer cells. We thus analyzed the tumorsphere formation of these cells. Strikingly, knockdown of PAR1 expression suppressed tumorsphere formation in MDA-MB-231 and Hs578T cells, as shown that the knockdown of PAR1 expression resulted in a much smaller size and lower density of tumorspheres than vector control (Fig. 5a). We noticed that vorapaxar also significantly suppressed tumorsphere formation in T47D-Twist and MDA-MB-231 cells (Supplementary Fig. 4a). Because breast CSCs are characterized by the CD44^high^/CD24^low^ phenotype^39,40^, we first determined the potential effect of Twist on cell population with CD44^high^/CD24^low^ properties by flow cytometry analysis. Indeed, Twist expression significantly increased the percentage of CD44^high^/CD24^low^ population in T47D cells (Fig. 5b). We then evaluated the effect of PAR1 on cell population with CD44^high^/CD24^low^ phenotype, showing that knockdown of PAR1 expression significantly reduced the percentage of CD44^high^/CD24^low^ population in MDA-MB-231 and Hs578T cells (Fig. 5b).

We also investigated the effect of PAR1 expression on the in vitro tumorigenicity using soft agar assay, showing that knockdown of PAR1 expression caused a remarkable decrease of colonies in MDA-MB-231 and Hs578T cells (Fig. 5c). We further tested the in vivo tumorigenicity in tumor xenograft experiments in which female SCID mice were injected with MDA-MB-231 and SUM159 cells with stable control vector or knockdown of PAR1 expression. Compared with vector control cells, MDA-MB-231 and SUM159 cells with stable knockdown of PAR1 expression resulted in dramatically reduced tumor growth in vivo (Fig. 5d–f). These data indicate that PAR1 plays critical roles in maintaining CSC properties and promoting tumorigenicity of breast cancer cells in vitro and in vivo.

### Inhibition of PAR1 suppresses metastasis of breast cancer

**Fig. 6:**
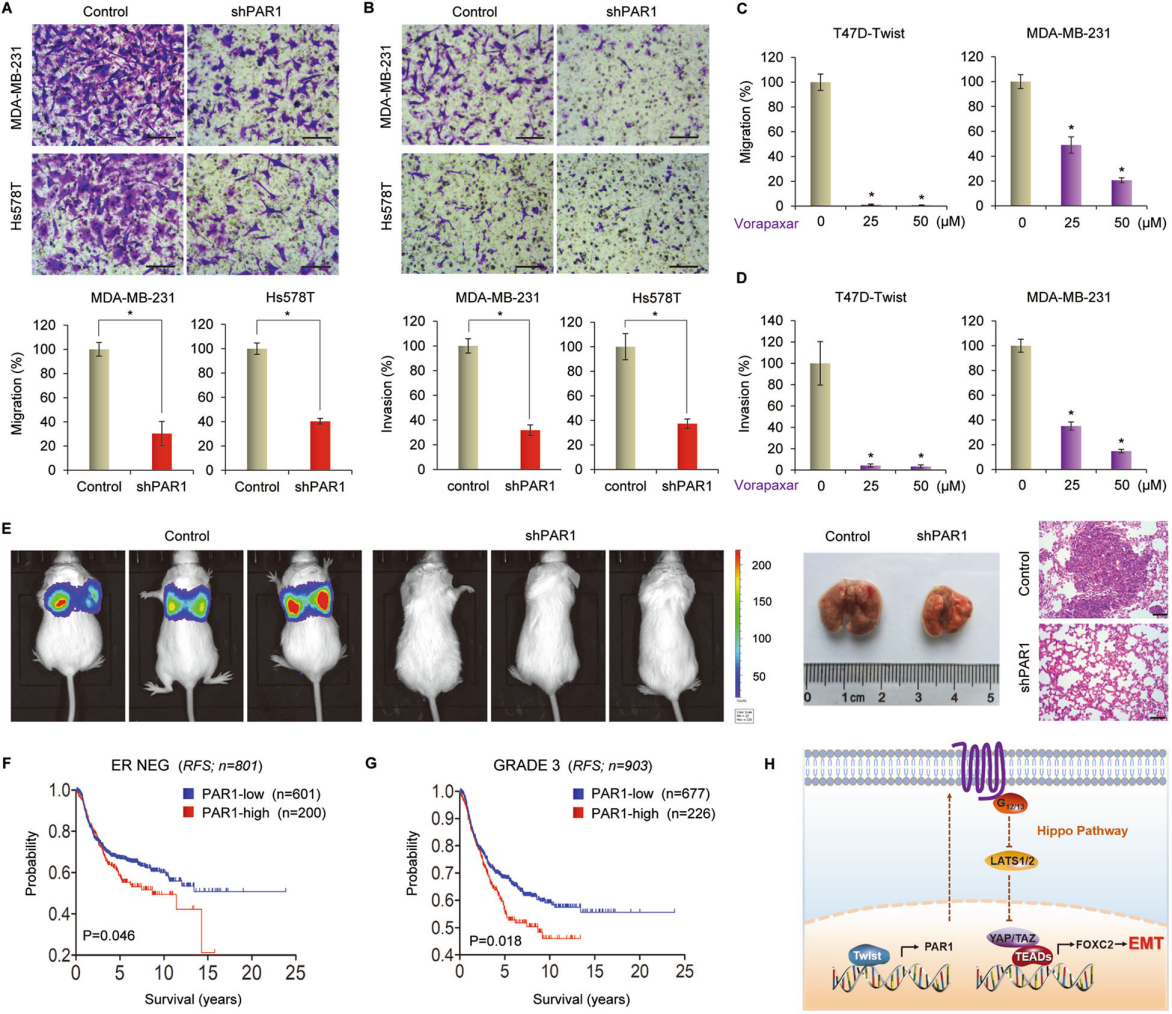
Inhibition of PAR1 suppresses metastasis in vitro and in vivo and elevated PAR1 predicts poor survival. **a** Migratory ability of MDA-MB-231 and Hs578T cells with stable control vector or knockdown of PAR1 expression was analyzed by transwell migration assay. The percentage of migratory cells is shown in the bar graphs. Data are mean ± SD of three separate experiments. *P < 0.01 by Student’s *t* test. Scale bars, 100 μm. **b** Invasiveness of MDA-MB-231 and Hs578T cells with stable control vector or knockdown of PAR1 expression was analyzed by transwell invasion assay. The percentage of invasive cells is shown in the bar graphs. Data are mean ± SD of three separate experiments. *P < 0.01 by Student’s *t* test. Scale bars, 100 μm. **c** Migratory ability of T47D-Twist and MDA-MB-231 cells treated with the indicated concentration of vorapaxar was analyzed by transwell migration assay. The percentage of migratory cells is shown in the bar graphs. Data are mean ± SD of three separate experiments. *P < 0.01 by Student’s *t* test. **d** Invasiveness of T47D-Twist and MDA-MB-231 cells treated with the indicated concentration of vorapaxar was analyzed by transwell invasion assay. The percentage of invasive cells is shown in the bar graphs. Data are mean ± SD of three separate experiments. *P < 0.01 by Student’s *t* test. **e** MDA-MB-231 cells with stable control vector or knockdown of PAR1 expression were injected into SCID mice via the tail vein. After 4 week, the development of lung metastases was recorded using bioluminescence imaging. Three representative mice from each group are shown (left panel). Lung metastatic nodules were examined in paraffin-embedded sections stained with hematoxylin and eosin (right panel). Scale bars, 100 μm. **f**, **g** Kaplan–Meier survival analysis for RFS of patients with ER negative (**f**) or Grade 3 (**g**) breast cancer in an aggregate breast cancer dataset according to PAR1 expression status. The *P*-value was determined by the log-rank test. **h** A proposed model to illustrate the transcription activation of PAR1 by Twist, which inhibits the Hippo signaling pathway in invasive breast cancer cells (see “Discussion”).

Increasing evidence shows that CSCs are highly tumorigenic and metastatic^41,42^. We further evaluated the effect of PAR1 on metastasis. Because loss of E-cadherin function can promote cell migration and invasion^43,44^ and because PAR1 inhibits E-cadherin expression, we hypothesized that PAR1 may play a crucial role in breast cancer cell migration and invasion. To test this notion, we conducted migration and invasion assays to assess the effect of PAR1 on cell migration and invasiveness. As anticipated, knockdown of PAR1 expression dramatically reduced the migratory and invasive capabilities of MDA-MB-231 and Hs578T cells in vitro (Fig. 6a, b). Vorapaxar also remarkably inhibited the migration and invasion of T47D-Twist and MDA-MB-231 cells (Fig. 6c, d and Supplementary Fig. 4b, c). Given the intimate link of PAR1 expression with breast cancer cell migration and invasion, we then assessed the effect of PAR1 expression on tumor metastasis in a xenograft metastasis model in which MDA-MB-231 cells with stable vector control and knockdown of PAR1 expression were injected via tail vein to generate pulmonary metastases. Remarkably, knockdown of PAR1 expression suppressed lung metastasis in vivo (Fig. 6e). These findings confirm that PAR1 is critical for metastasis of breast cancer cells.

Having identified the pivotal roles of PAR1 in breast cancer, we then determine the clinical relevance of PAR1 with patient survival in an aggregate breast cancer dataset. Kaplan–Meier survival analysis showed that high PAR1 expression exhibited shorter relapse-free survival (RFS) in ER-negative and Grade 3 breast cancer that are more aggressive than ER-positive and low-grade breast cancer (Fig. 6f, g). These data support the crucial roles of PAR1 in breast cancer aggressiveness.

## Discussion

In this study, we report that high PAR expression is correlated with invasive breast cancer, and PAR1 enhances the tumorigenic and metastatic capacity of breast cancer cells by attenuating the Hippo pathway and activating EMT program. Our study reveals new insights into the critical roles of PAR1 in EMT and breast cancer progression.

It has been documented that EMT plays key roles in cancer invasion and distant metastasis^2,4^. Loss of E-cadherin, a hallmark of EMT, has a tight correlation with tumor stage and metastasis^3^. Several transcription factors have been identified as EMT inducer, including Snail, Slug, Twist, ZEB1, and ZEB2^3,45–47^. Twist, a basic helix-loop-helix transcription factor, has a vital role in inducing EMT by downregulating E-cadherin expression in multiple types of invasive cancer cells^46,48^. Ectopic expression of Twist in breast cancer cells is sufficient to induce EMT, cell migration and invasion, and CSC-like properties, while knockdown of Twist expression suppresses invasion and metastasis of tumor cells^9,46,49^. Evidence has shown that apart from E-cadherin, Twist also possesses other molecular targets and downstream signaling pathways^50^. Our previous study and other’s findings have reported that Twist directly activates WNT5A and PDGFRα expression to promote tumor aggressiveness and metastasis^33,51^, and inhibits the p53 and Rb tumor suppressor pathways to override oncogene-induced premature senescence^52^. Recent research has shown that EMT can be induced by thrombin^53^. As a thrombin receptor, PAR1 is overexpressed in a range of malignant tumors, transmit signals in response to tumor-generated proteases and promote tumor progression^54^. PAR1 signaling can be activated not only by thrombin but also by tissue factor and matrix metalloproteases (MMPs)^21,55,56^. It has been reported that MMP-1 directly cleaves PAR1 for receptor activation and generates PAR1-dependent Ca^2+^ signals and migration^21^. Here, our data demonstrated Twist-mediated transactivation of PAR1, and showed a positive correlation between Twist and PAR1 expression, supporting that Twist is a direct transcriptional activator responsible for high PAR1 expression in highly invasive breast cancer and promotes tumor progression via PAR1 upregulation.

PAR1 activation triggers signaling through multiple heterotrimeric G proteins, including the Gq/11, Gi/o, and G12/13^57^. Recent research shows that PAR1 modulates Hippo pathway kinase Lats1/2 via G12/13 and Rho GTPase^19^. Consistent with this notion, PAR1 downregulation activated the Hippo pathway and inhibited YAP and TAZ expression, indicating the critical role of PAR1 in suppressing the Hippo pathway and thus triggering YAP/TAZ activation in invasive breast cancer cells. Activation of Foxc2 is closely associated with the induction of EMT and highly aggressive breast cancer^58^. Interestingly, we found that Foxc2 expression was significantly reduced in cells with PAR1 downregulation, indicating the involvement of PAR1 in Foxc2 expression and EMT induction via the Hippo signaling pathway. Together, our study demonstrated that Twist-mediated PAR1 expression contributed to YAP/TAZ and Foxc2 activation via suppressing the Hippo pathway, thereby inducing the EMT in breast cancer cells (Fig. 6h).

Extensive studies have shown that EMT induces a CSC-like phenotype that is the prerequisite for tumor initiation and metastasis^2,59^. Indeed, invasive breast cancer cells possess more CSC properties than non-invasive breast cancer cells^40,60^. In line with this notion, PAR1 expression results in the activated EMT program and the increased CSC properties in invasive breast cancer cells, indicating crucial roles of PAR1 in controlling the viability of CSCs. Inhibition of PAR1 in highly invasive breast cancer cells strongly suppressed tumorigenicity and metastasis both in vitro and in vivo. in addition, we demonstrated that elevated PAR1 expression occurred specifically in highly invasive breast cancer cells and predicted poor survival in highly invasive ER-negative and high-grade breast cancer patients. Our study clearly supports the critical roles of PAR1 in enhancing the aggressive behaviors of breast cancer cells.

Treatment of invasive breast cancer remains a major medical challenge because of few treatment options and poor response to conventional chemotherapy. Identification of the potential therapeutic targets may pave the way for highly effective cancer therapies. Our study identified the critical roles of PAR1 in EMT induction and maintenance of CSCs properties of invasive breast cancer cells, and knockdown of PAR1 expression efficiently reverted these functions, implying that PAR1 may be a potentially valuable molecular target for treating invasive breast cancer.

In summary, our findings highlight the importance of PAR1 in tumorigenicity and metastasis of breast cancer cells. We have shown that PAR1 as a direct transcriptional target of Twist, can promote EMT, migration, invasion, tumorigenicity, and lung metastasis of breast cancer cells through suppressing the Hippo pathway, providing a potential therapeutic target for the treatment of invasive breast cancer.

## Supplementary information

Supplementary Figure Legends

Supplementary Figure 1

Supplementary Figure 2

Supplementary Figure 3

Supplementary Figure 4

## Data Availability

The microarray datasets that were utilized in the study were retrieved from NIH-GEO dataset database (http://www.ncbi.nlm.nih.gov/gds/), including: GSE12777, GSE10890, GSE16732, GSE24202 and GSE53222, and EMBL-EBI dataset database (https://www.ebi.ac.uk/), including E-TABM-157 and E-MTAB-181. Information about TCGA, the investigators and institutions that constitute TCGA research network can be found at http://cancergenome.nih.gov/. Other data generated or analyzed during this study are included in this published article.
