# Supplementary material for: Twist-mediated PAR1 induction is required for breast cancer progression and metastasis by inhibiting Hippo pathway
Source: Cell Death Dis. 2020 Jul 9;11(7):520. doi: 10.1038/s41419-020-2725-4 (PMC7347637; doi:10.1038/s41419-020-2725-4)
Supplement: Supplementary file 1 — Supplementary Figure Legends [file 41419_2020_2725_MOESM1_ESM.docx]

**Supplementary Figure Legends**

**Supplementary Figure 1. Elevated expression of PAR1 highly correlated with invasive breast cancer cells.**

**a** Box plots and bar charts indicated PAR1 mRNA expression in different invasion abilities of breast cancer cells from four gene expression datasets (E-TAMB-157, GSE16732, GSE12777 and GSE10890).

**b** Expression of PAR1 mRNA was analyzed by quantitative real-time PCR in a representative panel of breast cancer cell lines (MDA-MB-231 is abbreviated to MDA231). Data are shown as mean ± SD based on three independent experiments.

**Supplementary Figure 2. Twist inhibits the Hippo pathway via PAR1.**

**a** Expression of YAP and TAZ was examined by Western blotting in T47D cells transfected with control vector or Twist-expressing vector, and actin was served as a loading control. Representative images were presented from three independent experiments.

**b** Expression of CTGF, ANKRD1, Cyr61, and BIRC5 was analyzed by quantitative real-time PCR in T47D cells transfected with control vector or Twist-expression vector. Data are presented as mean ± SD of three separate experiments, *, *P*<0.01 by Student’s *t* test.

**c** Expression of Twist, PAR1, YAP, and TAZ was analyzed by western blot in Hs578T cells with stable control vector or knockdown of Twist expression, and actin was served as a loading control. Representative images were presented from three independent experiments.

**d** Expression of TAZ was analyzed by quantitative real-time PCR in MDA-MB-231 and Hs578T cells with stable control vector or knockdown of PAR1 expression. Data are presented as mean ± SD of three separate experiments.

**Supplementary Figure 3. TRAP6 activates YAP/TAZ through Rho.**

**a** T47D-Twist and SUM159 cells were serum-starved for 24 h and then stimulated with Thrombin at the indicated doses (units per milliliter) for 30 min. The cells were then lysed and subjected to Western blotting analysis with the indicated antibodies. Representative images were presented from three independent experiments.

**b** T47D-Twist cells were serum-starved for 24 h, pretreated with 2 μg/mL C3 for 4 h and then stimulated with 2 μM TRAP6 for 1h. Cells were lysed and subjected to Western blotting analysis with the indicated antibodies. Representative images were presented from three independent experiments.

**Supplementary Figure 4. Pharmacological inhibition of PAR1 suppresses tumorsphere formation, migration and invasion of breast cancer cell.**

**a** Tumorsphere formation was analyzed in T47D-Twist and MDA-MB-231 cells treated with the indicated concentration of vorapaxar (bottom panel). Representative images of tumorspheres were shown (upper panel). Scale bars, 100 μm. Data are presented as a percentage of control cell lines. #, *P* < 0.05 and *, *P* < 0.01 by Student’s *t* test.

**b** Migratory ability of T47D-Twist and MDA-MB-231 cells treated with the indicated concentration of vorapaxar was analyzed by transwell migration assay. Representative images were presented from three separate experiments. Scale bars, 200 μm.

**c** Invasiveness of T47D-Twist and MDA-MB-231 cells treated with the indicated concentration of vorapaxar was analyzed by transwell invasion assay. Representative images were presented from three separate experiments. Scale bars, 200 μm.
