# Supplementary figures and images for: Twist-mediated PAR1 induction is required for breast cancer progression and metastasis by inhibiting Hippo pathway
Source: Cell Death Dis. 2020 Jul 9;11(7):520. doi: 10.1038/s41419-020-2725-4 (PMC7347637; doi:10.1038/s41419-020-2725-4)

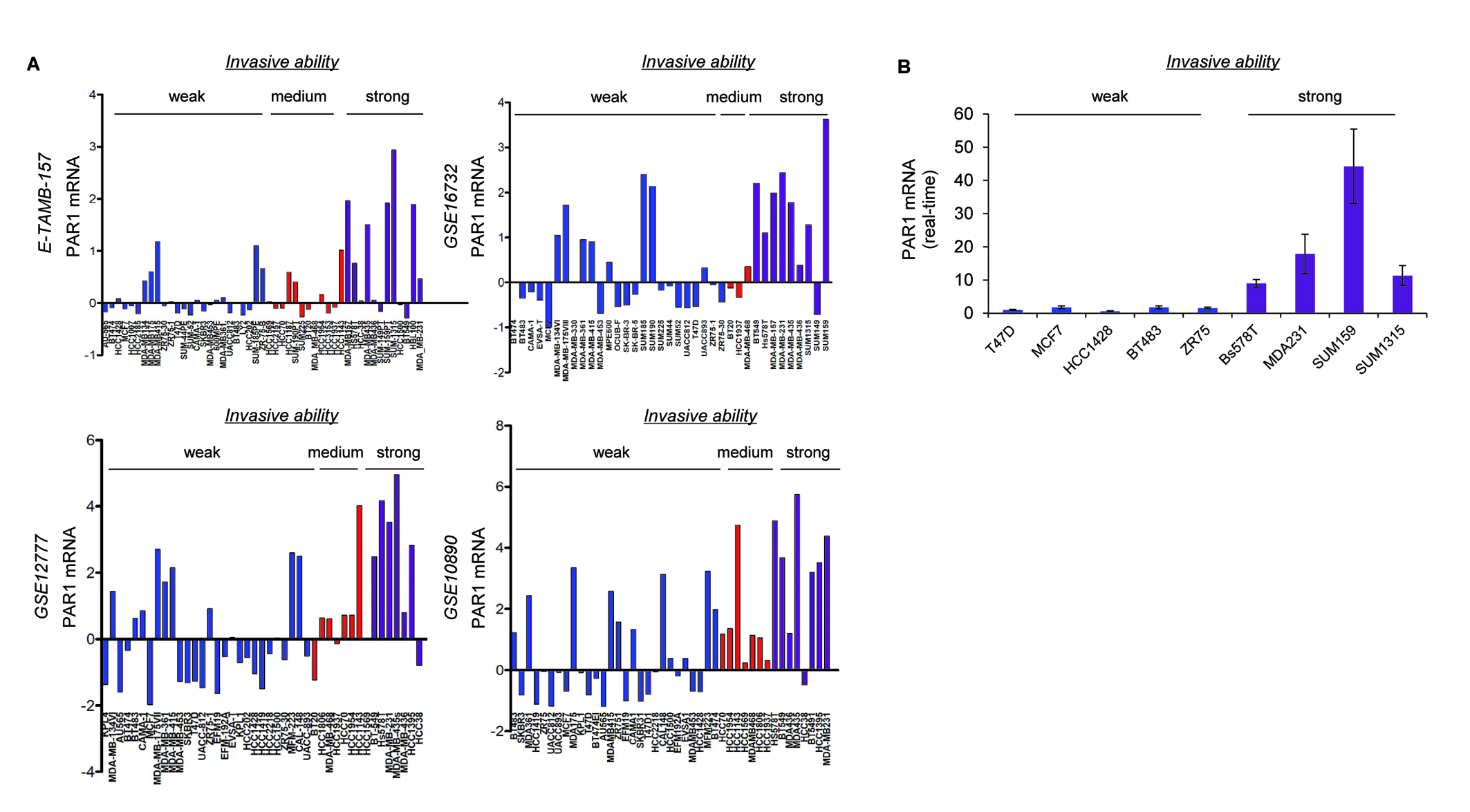

Supplement: Supplementary file 2 — Supplementary Figure 1 [file 41419_2020_2725_MOESM2_ESM.tif]

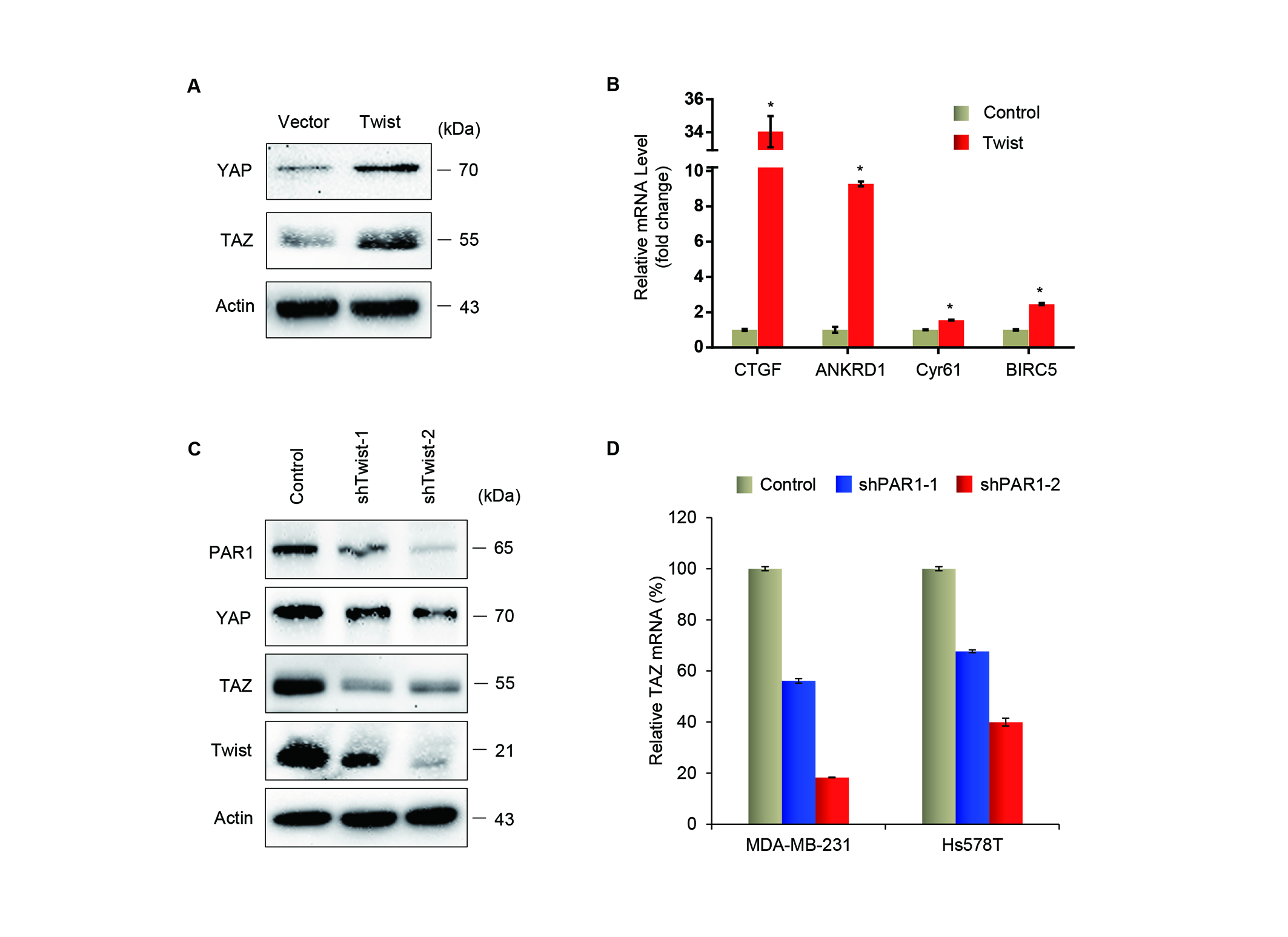

Supplement: Supplementary file 3 — Supplementary Figure 2 [file 41419_2020_2725_MOESM3_ESM.tif]

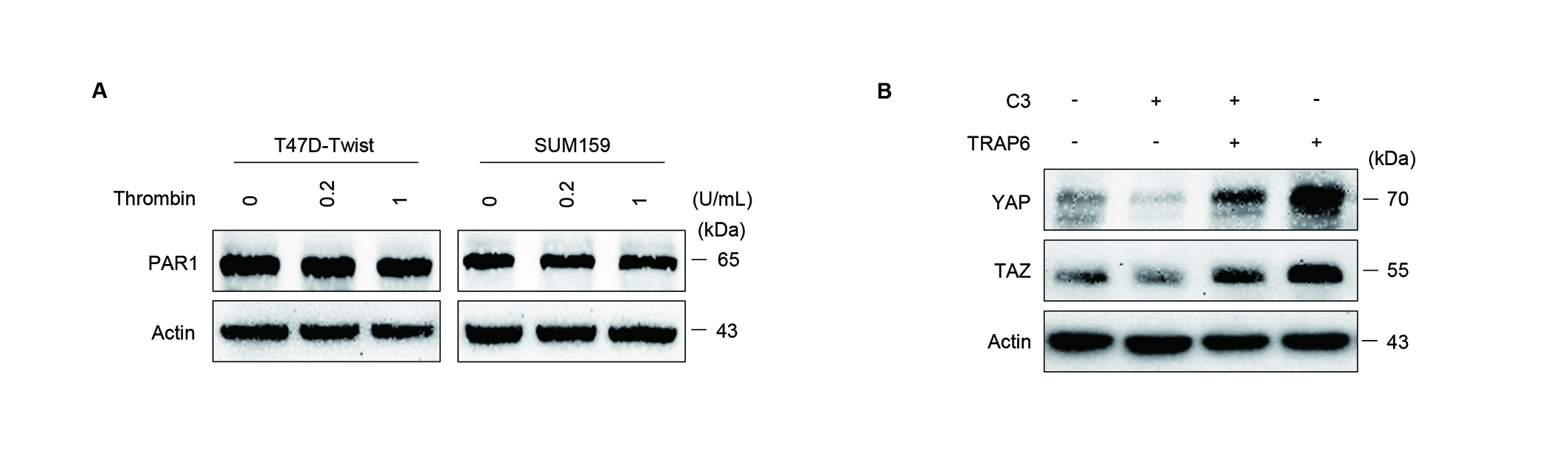

Supplement: Supplementary file 4 — Supplementary Figure 3 [file 41419_2020_2725_MOESM4_ESM.tif]

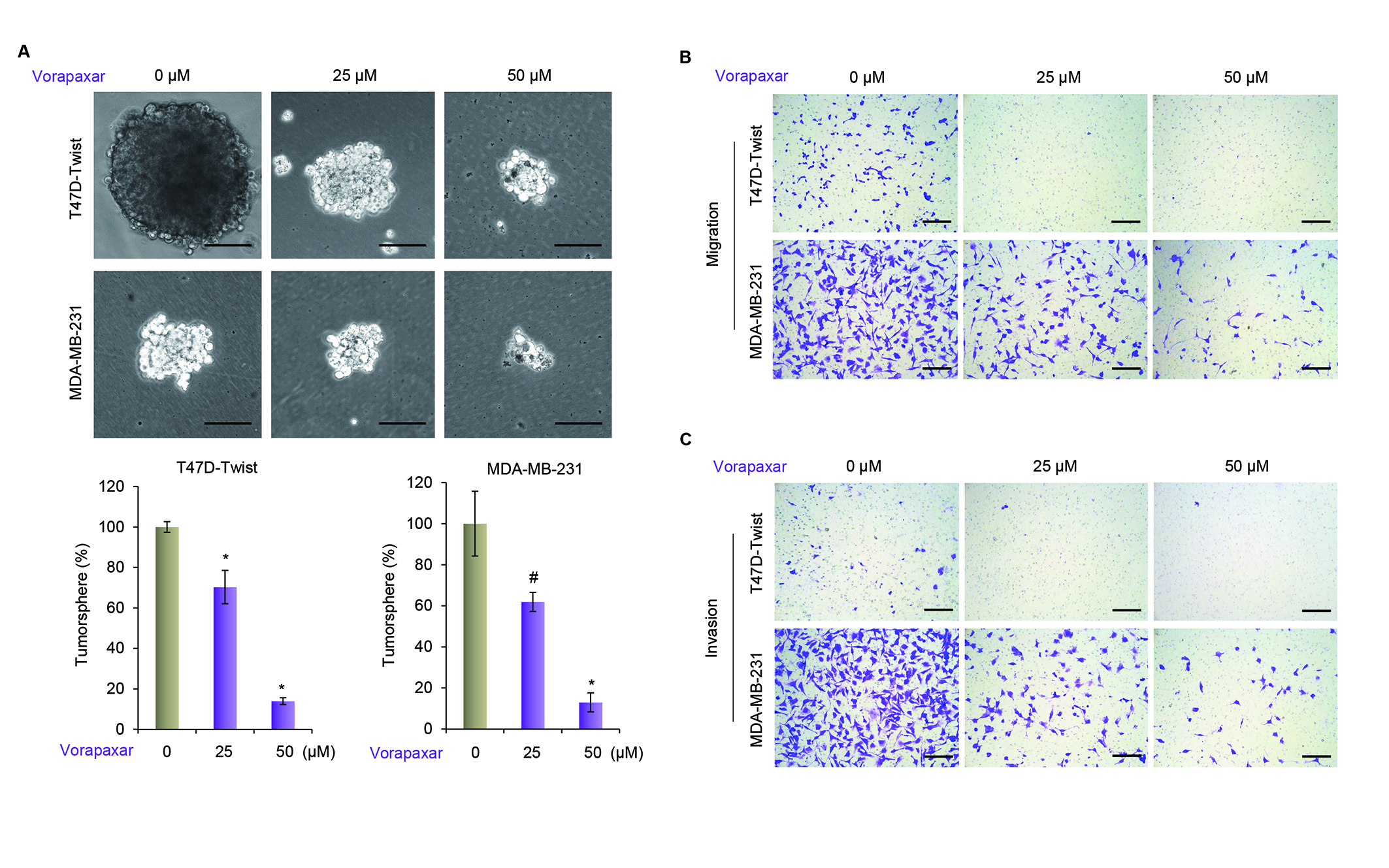

Supplement: Supplementary file 5 — Supplementary Figure 4 [file 41419_2020_2725_MOESM5_ESM.tif]
